# Domestic Summary

**Published:** 1840-02-29

**Authors:** 


### DOMESTIC SUMMARY.

*Difficulty of Diagnosis of the Injuries of the Hip.* (Extracted from Dr. Norris' Report of Surgical Cases treated in the Pennsylvania Hospital.)

Three cases of faulty diagnosis in affections of the hip came under notice during the term. The first was a case of coxalgia, mistaken for luxation, in a patient ætat. twelve, who was brought to us from a neighbouring state. A short time previously, he had been suddenly attacked with lameness in the right limb, which was attributed by his friends to some injury received a short time before in play. Two physicians, who had been called in to see the boy, pronounced him to be labouring under dislocation of the hip, and had made two strong efforts with the pullies to reduce it; but after causing great suffering, they gave up all hope of replacing the bone, and sent him to Philadelphia. The symptoms were all those of disease of the hip joint, in its early stage. The attitude was that assumed by those labouring under coxalgia. The left limb was apparently lengthened, though accurate examination, with the patient placed on his back, and the spinous processes on a line with each other, showed the limbs to be of equal length; the appearance of lengthening being produced solely by an inclination of the spine to that side; the left buttock was flattened, and the motions of the joint were found to be perfect, though executed by the patient with extreme caution, and causing some pain.

The second case was that of a healthy boy, presented for admission, who had been confined to bed, and treated during some months, for fracture of the neck of the thigh bone. His injury had been produced by an empty wagon passing over his limb. Upon examination, his right limb was found to be about two and a half inches shorter than that of the opposite side; the head of the bone could be traced on the ileum, the toes were inclined inwards, and it was clearly perceptible that a dislocation upwards and backwards existed. No pain was produced by the handling of the limb, nor was inflammation, swelling, or any other symptom of disease about the parts present.

The third case happened in our own practice, and was that of a man, ætat. thirty-five, admitted with an injury of the hip, produced by a fall from a height upon the part. Great pain was suffered in any attempt to move the limb; but the usual evidences of fracture of the neck of the bone were absent, and the case was looked upon as one of severe contusion. After some days, however, eversion of the toes and shortening of the limb, which was found to have occurred, left no doubt as to the true nature of the accident. Desault's apparatus was now applied, and the limb drawn down to its natural length. Slight ulceration of the heel, however, followed, and prevented a sufficient degree of extension from being kept up, and the patient will have some shortening of the limb.

Although the signs by which luxations and fractures of the neck of the thigh bone may be distinguished from each other, and from simple contusions and diseases of this part, are dwelt upon in all treatises on surgery, and are made out to be readily distinguishable and well marked, yet all practical surgeons are aware of the difficulties of diagnosis sometimes attendant upon the various injuries about the hip upon actual inspection. The true nature of the injury in these cases is often more evident some hours or days after the receipt of the accident, than immediately after its occurrence; and I am inclined to think that the necessity of close *secondary* examinations, in all instances in which there is room for a doubt as to the nature of the injury, are not sufficiently insisted on. Had careful examinations of the first two of the above cases been made the day following the receipt of the injuries, it is hardly possible that their true natures would have been mistaken; and the symptoms of fracture in the third case were only detected after a lapse of some days, though this injury was at first suspected, and the patient attentively examined. It is, however, more particularly where fractures may be suspected that these repeated examinations are demanded.

The following cases of fracture of the hip, which I witnessed a few years ago, are interesting, in connexion with those just given, on account of the difficulty of diagnosis, and are well calculated to show the necessity of close and repeated examinations in all injuries of this part.

John Henrick, ætat. fifty-two, was admitted, November 27th, 1831, for an injury of the hip received by falling down a few steps. He complained of excessive pain about the joint, and was unable to rise, or in any way use the limb. Accurate measurement from the anterior superior spinous process to the internal malleolus, showed the limb to be of the natural length. No deformity existed about the joint, no crepitus could be detected, and the toes were not thrown outwards. The injury was looked upon as a contusion only; and rest, and the application of cups, were the remedies prescribed.

On the fifth day after his admission he had a severe attack of mania-a-potu; and, during his delirium, was out of bed, stood upon and moved his limb considerably. Having recovered from this attack, he was sent back from the room to which he had been removed, to the surgical ward; and it was then found that the limb was shortened a full inch and a half, and the knee and toes everted, though a daily examination of the limb up to the time of this showed nothing amiss about it. The case was now supposed to be one of dislocation; but, upon accurate examination, was discovered to be a fracture through the great trochanter.

In January, 1831, another case, very similar to the above, occurred in a patient ætat. thirty-eight. When admitted, he had great pain in the hip, but the toes were not everted; no crepitus could be detected; and, on accurate measurement, no shortening of the limb was observable. A short time after his entrance, he also was attacked with mania-a-potu; and, on recovery, shortening was discovered. Desault's splints were applied with the effect of counteracting, in a measure, the shortening; and the man left the hospital on the 20th of April, walking with a stick.

The exact length of the limb on entrance, with the natural position of the foot, and absence of any deformity, or crepitus, led to the supposition, in both of the above cases, despite the great pain suffered, that simple contusions only of the part existed, and this idea was confirmed at the commencement of the attacks of mania-a-potu, upon seeing the men up and moving about the room; but, after recovery from their attacks, the eversion of the foot led to an immediate examination, when the shortening was found to exist, which, in connexion with the symptom just mentioned, could only be caused by fracture of the neck of the bone. In both cases the fragments must have been interlocked in such a way as to have prevented any shortening or motion, and so remained till the delirium occurred, when the violent efforts made to use the limb, unlocked the parts, and permitted the lower fragments to be drawn upwards.

Previously to witnessing these cases we had believed it impossible for a patient with fracture of the neck of the femur to walk upon the limb; but upon examining the records of our science on this point, I find that similar instances are noted. Sabatier, in tom. 4 of the *Mémoires de l'Acad. de Chirurg.*, has recorded an instance in which the patient walked home, and even got up the next morning, after an injury of this kind. Desault states that he has seen similar cases. Boyer says that he saw a man who was able to walk, with the aid of a stick, during several days after a like accident. Dr. M'Tyer, in the 4th vol. of the *Glasgow Medical Journal*, details a case of injury of the hip, which had not confined the patient from her usual occupations, but which was proved upon dissection, three months after, to be a case of fracture within the capsular ligament.

Since the occurrence of the cases I have described, Mr. Syme, in the *Edin. Med. and Surg. Journ.* for 1836, and M. Malle, in his *Clinique Chirurgicale*, have each given a case of fractured neck of the thigh, in which the patients walked some days after it; and I have myself seen another hospital patient, with a similar fracture, (proved by post mortem examination,) who assured me that he had walked some squares after the occurrence of his accident.

*Amer. Journ. of Med. Sciences.*

---

*A Case of Stricture of the Urethra cured by Bougies of Bark of the Slippery Elm Tree.* By Wm. Waters, M. D., of Fredericktown, Md.—Mr. ——, ætat. about 28, of strumous habit and ill health, informed me that he was threatened with retention of urine from stricture. According to his history of the case, he had laboured under blennorrhœa for *thirteen* years; for the last *three* years symptoms of stricture existed at times, and for the last twelve months his urine always flowed in a forked stream, or twisted like a cork-screw, evidently denoting permanent stricture. For the last year past he had been using the gum elastic bougie for the twofold purpose of relieving the discharge as well as the stricture. On the 14th of August last I was consulted, when I found him labouring under considerable local inflammation, with purulent discharge, and great difficulty in urinating. By leeching along the course of the urethra, and other antiphlogistic means, the active inflammation was reduced in a few days. I then examined the stricture with a bougie, and found it seated about four inches from the glans penis, and admitting only the smallest size gum elastic bougie. I immediately put him on the use of bougies prepared from the bark of slippery elm. They were made very smooth, and of gradual increase of size, shaped like the ordinary gum elastic. Before using, they were held a few moments in tepid water, and were permitted to remain in the urethra generally half an hour, sometimes an hour or more. On one occasion, when introduced at bed time, he fell asleep with it in, and rested well all night, suffering not the least inconvenience whatever. With the use of these my patient was delighted; their soothing properties were brought into strong contrast with the pain from the wonted use of the gum elastic. In one week it was necessary to enlarge the bougies by glueing the smooth sides of the bark together. By their use for three weeks with comparatively no pain, the urethra was enlarged to the ordinary calibre. In urinating for the last three months the stream has flowed full and without impediment. In the mean time the discharge diminished as the stricture was dilated. It will be recollected that the gum elastic in this case had been used not only without improvement, but with an aggravation of the stricture. The comparative merits of the elm bougie over other bougies in ordinary use I will leave to the future test and experience of the profession. But in certain cases of stricture where the more potent are not immediately wanted, I should prefer the emollient and expansive qualities of the elm over the wooden, gum elastic, whale bone, ivory, or metallic. For the introduction of the elm bougie to the notice of the profession, I am indebted to an interesting account of the Bark of the Slippery Elm Tree, (*Ulmus fulva,*) for Bougies, Tents and Catheters, by William A. McDowell, M. D., of Fincastle, Virginia, published in the Western Journal of Med. and Phys. Sciences, Jan. 1838.

*Fredericktown,* December 10th, 1839.

*Am. Journ. Med. Sciences.*

